# Optimal Power and Efficiency of Multi-Stage Endoreversible Quantum Carnot Heat Engine with Harmonic Oscillators at the Classical Limit

**DOI:** 10.3390/e22040457

**Published:** 2020-04-17

**Authors:** Zewei Meng, Lingen Chen, Feng Wu

**Affiliations:** 1 Institute of Thermal Science and Power Engineering, Wuhan Institute of Technology, Wuhan 430205, China; mengzw94@163.com (Z.M.); 13006338568@163.com (F.W.); 2 School of Mechanical & Electrical Engineering, Wuhan Institute of Technology, Wuhan 430205, China; 3 College of Power Engineering, Naval University of Engineering, Wuhan 430033, China

**Keywords:** finite time thermodynamics, quantum Carnot heat engine, combined cycle, harmonic oscillator system, power, efficiency

## Abstract

At the classical limit, a multi-stage, endoreversible Carnot cycle model of quantum heat engine (QHE) working with non-interacting harmonic oscillators systems is established in this paper. A simplified combined cycle, where all sub-cycles work at maximum power output (MPO), is analyzed under two types of combined form: constraint of cycle period or constraint of interstage heat current. The expressions of power and the corresponding efficiency under two types of combined constrains are derived. A general combined cycle, in which all sub-cycles run at arbitrary state, is further investigated under two types of combined constrains. By introducing the Lagrangian function, the MPO of two-stage combined QHE with different intermediate temperatures is obtained, utilizing numerical calculation. The results show that, for the simplified combined cycle, the total power decreases and heat exchange from hot reservoir increases under two types of constrains with the increasing number (N) of stages. The efficiency of the combined cycle decreases under the constraints of the cycle period, but keeps constant under the constraint of interstage heat current. For the general combined cycle, three operating modes, including single heat engine mode at low “temperature” (SM1), double heat engine mode (DM) and single heat engine mode at high “temperature” (SM2), appear as intermediate temperature varies. For the constraint of cycle period, the MPO is obtained at the junction of DM mode and SM2 mode. For the constraint of interstage heat current, the MPO keeps constant during DM mode, in which the two sub-cycles compensate each other.

## 1. Introduction

In order to obtain a more practical method for analyzing thermodynamic processes under the condition of finite time and limited scale, finite time thermodynamics (FTT) has been developed from classical thermodynamics and has been a branch of modern thermodynamics [1,2,3,4,5,6,7,8,9,10,11,12,13,14,15,16,17,18,19,20].

By applying FTT theory, scholars have conducted extensive research on one-stage thermodynamic cycles with traditional working medium (WM), and have gradually focused on multi-stage combined cycles in classical heat engine (HE). In 1982, Rubin and Andresen [21] first attempted to investigate composite systems in finite-time thermodynamics. The two-stage combined classical Carnot HE with intermediate heat reservoir was analyzed and optimized. The results showed that the overall efficiency of the combined HE was still equal to CA efficiency. When one of the combined HE deviated from MPO, the other would exactly compensate for this to make the combined HE operate at MPO. Then, Chen and Yan [22] optimized a multi-stage combined endoreversible HE without an intermediate heat reservoir and obtained a relationship between the efficiency and output power. Wu [23] and Wu et al. [24] studied the optimal power of a combined power cycle without any intermediate heat reservoirs. By cascading different cycles and changing WM, the total available temperature range could be expanded. The overall efficiency of the combined HE could be improved over that of a single-stage HE. Chen et al. [25,26,27] optimized the specific power [25,26] and analyzed the effect of heat leakage [27]. By introducing an irreversible factor from references [28,29,30,31,32], Chen et al. [33] established a class of more generalized irreversible combined power plant cycle model. Utilizing FTT and entransy theory, Cheng [34] also optimized multi-stage combined Carnot HE under three types of objectives (power, efficiency, and thermo-economic performance). By introducing the entransy transfer efficiency, Wu [35] developed an effective method to calculate the operating temperatures of sub-cycle in multi-stage combined cycle. The above research results contributed to revealing the energy conversion laws in the classical system.

In fact, FTT theory has also been used to study the one-stage cycle in quantum heat engine (QHE). In 1984, Kosloff [36] studied a QHE model, using a harmonic oscillators system as the WM. By solving the time evolution equation of the system, reference [36] obtained the expressions of the output power and efficiency of QHE. In 1992, Geva and Kolsoff [37], combining FTT and quantum mechanics for the first time, systematically studied the output performance of the QHE working with spin-1/2 under finite thermal resistance. In the same year, Geva and Kosloff [38] introduced a new WM, a harmonic oscillators system, and constructed an endoreversible Carnot QHE model. The performance parameters at the MPO at the classical limit were derived. Later, more WMs were considered in the QHE. In 2000, Bender et al. [39] constructed a reversible cycle of QHE, working with particles in an infinite square potential well. The expressions of output work, power, efficiency, and entropy generation at two-state and multiple-state were derived. Based on these models, when the well width moved at a low but finite speed, Abe [40] and Abe and Okuyama [41,42] further derived the MPO and efficiency using the FTT method. In 2001, ŞiŞman and Saygin [43,44,45,46] extended WMs to ideal Bose gases and ideal Fermi gases, and analyzed output work and efficiency of HE. The results showed that the Carnot efficiency is not affected by quantum degeneracy, but the cycle work depends on quantum degeneracy. In 2011, Wang et al. [47] studied on an analogous Carnot cycle. Assuming that the moving speed of potential well is very slow, the general efficiency, determined by the expectation value of Hamiltonian, was obtained and was similar to Carnot efficiency. Moreover, Abe [48] discussed the reversible Carnot-like HE and obtained the general expression of efficiency under an arbitrary potential well, which is determined by the specific shape of the potential well. It was pointed out that, compared with genuine classical Carnot HEs, this type of HE is generic but is not universal. Combining optimal control theory and FTT theory, Erdman et al. [49] optimized the two-level HE by a fast Otto-cycle and derived the closed formula of MPO and the corresponding efficiency. In addition, more genres of WM have been considered and studied recently [50,51,52,53,54,55,56]. To date, the research on quantum thermodynamic cycles has mainly focused on the optimal path and optimal performance in one-stage HEs, including Carnot HEs [37,38,39,44,55,56,57,58], Brayton HEs [59,60,61,62,63,64,65,66], Otto HEs [46,67,68,69,70,71,72,73,74,75,76], Stirling HEs [45,77,78,79,80,81], and other HEs and systems [43,82,83,84,85,86,87]. Different optimization objects and different WMs, from endoreversible to irreversible QHE cycles, were also focused on; see the review articles [88,89,90,91,92].

For multi-stage heat engines, it can operate in parallel, in tandem and in other forms. The multi-stage heat engine operating in parallel can enhance the output power in the fixed temperature range. The one operating in tandem can enhance output power and energy utilization by enlarging temperature range. Miller et al. [93] studied the role of fluctuations in tandem configuration and developed a quantum geometric framework. The one in other forms, such as a QHE which is a strongly coupled open quantum system, was studied recently by Campisi et al. [94].

There is no work performed for the combined cycle of QHE with FTT theory in the open literature. In this paper, the FTT theory will be employed to investigate the output characteristics of combined QHE. Based on references [21,38], this paper will establish a combined cycle model of QHE operating in tandem by taking non-interacting harmonic oscillators at the classical limit as WM. A simplified combined cycle and a general combined cycle will be investigated, respectively. The optimal relationship of output performance and the corresponding operation parameters will be derived and analyzed.

## 2. Theory Model in Quantum Regime

### 2.1. The Description Equation for Harmonic Oscillators

According to quantum mechanics and quantum statistics, the Hamiltonian of non-interacting harmonic oscillators is described in the following form [38,95]

(1) $${\hat{H}}_{S}=\hbar \omega \hat{N}=\hbar \omega {\hat{a}}^{+}\hat{a}$$

where $\hbar =1.05\times 10^{-34}(J\cdot s)$ is denoted as the Dirac constant (reduced Planck’s constant), ω is denoted as the oscillator’s frequency, $\hat{N}={\hat{a}}^{+}\hat{a}$ is denoted as the number operator, and ${\hat{a}}^{+}$($\hat{a}$) is denoted as the Bosonic creation operator (annihilation operator).

The population of the harmonic oscillators is denoted as

(2) $$n=\langle \hat{N}\rangle =1/\left(e^{\hbar \omega /\left(k_{B}T\right)}-1\right)=1/\left(e^{\hbar \omega \beta }-1\right)$$

where $k_{B}=1.38\times 10^{-23}(J/K)$ is denoted as Boltzmann constant, and T is denoted as the absolute temperature in harmonic oscillators system, which is replaced by $\beta =1/\left(k_{B}T\right)$ in this paper.

Combined with Equations (1) and (2), the expectation value of the Hamiltonian of harmonic oscillators (i.e., the internal energy of the WM) is denoted as

(3) $$E_{S}=\langle {\hat{H}}_{S}\rangle =\hbar \omega (t)\langle \hat{N}\rangle =\hbar \omega (t)n$$

The variation in internal energy is denoted as

(4) $$dE_{S}=d\langle {\hat{H}}_{S}\rangle =\hbar nd\omega +\hbar \omega dn$$

Comparing the first law of thermodynamics in microscope system, the work and heat exchange in the quantum system is similarly defined as, respectively,

(5) dW=ℏndω

(6) dQ=ℏωdn

### 2.2. The Evolution Equation of an Observable

In the Heisenberg picture, the evolution of the arbitrary operator ( ) with time is determined by the quantum master equation (QME), that is

(7) $$\frac{d\hat{X}}{dt}=\frac{i}{\hbar }\left[{\hat{H}}_{S},\hat{X}\right]+\frac{\partial \hat{X}}{\partial t}+L_{D}(\hat{X})$$

where $L_{D}(\hat{X})$ is denoted as a dissipation term, originating from the coupling effect between the WM system and heat reservoirs [89]. Utilizing the semigroup theory, the dissipation term is given by

(8) $$L_{D}(\hat{X})=\sum_{\alpha }\gamma_{\alpha }({\hat{V}}_{\alpha }^{+}\left[\hat{X},{\hat{V}}_{\alpha }\right]+\left[{\hat{V}}_{\alpha }^{+},\hat{X}\right]{\hat{V}}_{\alpha })$$

where [ ] is Poisson brackets. For given arbitrary operators $\hat{x}$ and $\hat{y}$, its operational rule is $\left[\hat{x},\hat{y}\right]=\hat{x}\hat{y}-\hat{y}\hat{x}$. ${\hat{V}}_{\alpha }^{+}$ is Hermitian conjugate operator of ${\hat{V}}_{\alpha }$ in the Hilbert space, and $\gamma_{\alpha }$ is phenomenological positive coefficients.

Setting $\hat{X}=\hat{N}$, ${\hat{V}}_{\alpha }^{+}={\hat{a}}^{+}$, $V_{\alpha }=\hat{a}$, then using Equations (7) and (8) yields

(9) $$\dot{n}=d\langle \hat{N}\rangle /dt=\langle {\hat{L}}_{D}(\hat{N})\rangle =-2ae^{q\hbar \beta_{j}\omega }\left[(e^{\hbar \beta_{j}\omega }-1)n-1\right]$$

where a and q are constant parameters of heat reservoirs, meeting a>0 and 0>q>−1, respectively, and $\beta_{j}$ and ω are the temperature and phonon frequency of heat reservoirs, respectively.

### 2.3. The Process of Carnot Cycle

#### 2.3.1. Isothermal Branches

When the harmonic oscillators are coupled with heat reservoirs and its temperature keeps constant, the system undergoes an isothermal process. During the isothermal process, the “temperature” of the harmonic oscillators system is designated as $\beta^{\prime }$, and the population of harmonic oscillators varies from $n_{i}$ to $n_{f}$.

During the isothermal process, by integrating Equation (6), the amounts of heat exchange between WM and heat reservoir are obtained by

(10) $$Q_{if}=\hbar \int_{n_{i}}^{n_{f}}\omega dn=\int_{n_{i}}^{n_{f}}\frac{1}{\beta^{\prime }}\ln\left(\frac{1}{n}+1\right)dn=\frac{F\left(n_{i},n_{f}\right)}{\beta^{\prime }}$$

where $F\left(n_{i},n_{f}\right)=n_{f}\ln\left[(1+n_{f})/n_{f}\right]-n_{i}\ln\left[(1+n_{i})/n_{i}\right]+\ln\left[\left(1+n_{f})/(1+n_{i}\right)\right]$

The work is integrated along the isothermal process and is denoted as

(11) $$W_{if}=\hbar \int_{\omega_{i}}^{\omega_{f}}nd\omega =\frac{1}{\beta^{\prime }}\ln\frac{n_{i}}{n_{f}}+\hbar (\omega_{i}-\omega_{f})$$

For Equations (10) and (11), when $n_{f}>n_{i}$, the WM absorbs heat from heat reservoirs, the outside environment does positive work on harmonic oscillators system, and the integrals in Equations (10) and (11) are positive values. Otherwise, the integrals are negative values.

When coupled with heat reservoirs, the population of harmonic oscillators varies from $n_{i}$ to $n_{f}$. During the isothermal process, according to Equation (9), the consuming time is calculated by

(12) $$\tau =\int_{n_{i}}^{n_{f}}\frac{dn}{\dot{n}}=\int_{\ln\left(\left(n_{i}+1\right)/n_{i}\right)}^{\ln\left(\left(n_{f}+1\right)/n_{f}\right)}{\left[e^{q\alpha x}\left(e^{\alpha x}-e^{x}\right)\left(1-e^{-x}\right)\right]}^{-1}dx$$

where $\alpha =\beta /\beta^{\prime }$ and $x=\hbar \beta^{\prime }\omega$, q and β are the constant parameter and temperature of heat reservoir, respectively, and ω and $\beta^{\prime }$ are the frequency and the temperature of WM, respectively.

#### 2.3.2. Adiabatic Branches

During the adiabatic branches, the heat exchange between the harmonic oscillators system and heat reservoir is zero. Therefore, the output work along the adiabatic process is equal to the variation in internal energy. Ignoring the quantum non-adiabatic phenomenon, that is $n_{i}=n_{f}$. According to Equation (5), the work is denoted as

(13) $$W=-\Delta E_{S}=-\hbar \omega_{f}n_{f}+\hbar \omega_{i}n_{i}=\hbar \left(\omega_{i}-\omega_{f}\right)n_{f}$$

When $\omega_{f}<\omega_{i}$, the harmonic oscillators do positive work and Equation (13) is a positive value. Otherwise, the work is a negative value.

### 2.4. The One-Stage Endoreversible Carnot Cycle

**Figure 1:**
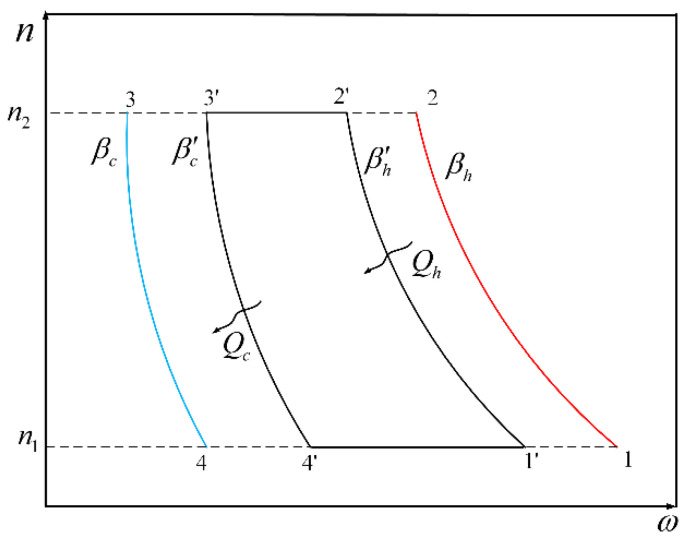
The diagram of one-stage Carnot cycle in quantum harmonic oscillators system.

The quantum Carnot cycle with harmonic oscillators system is shown in Figure 1, in which the cycle consisting of 1-2-3-4 processes is a reversible Carnot cycle and the cycle consisting of $1^{\prime }\text{-}2^{\prime }\text{-}3^{\prime }\text{-}4^{\prime }$ processes is an endoreversible Carnot cycle (existing thermal resistance between the WM and heat reservoir).

During the isothermal heating process ($1^{\prime }\text{-}2^{\prime }$ branch), according to Equation (10), the amount of absorbing heat is

(14) $$Q_{h}=Q_{1^{\prime }2^{\prime }}=\frac{F\left(n_{1},n_{2}\right)}{{\beta^{\prime }}_{h}}$$

According to Equation (12), the corresponding consumed time is given by

(15) $$\tau_{1^{\prime }2^{\prime }}=\int_{\ln\left(\left(n_{1}+1\right)/n_{1}\right)}^{\ln\left(\left(n_{2}+1\right)/n_{2}\right)}{\left[e^{q\alpha_{h}x_{h}}\left(e^{\alpha_{h}x_{h}}-e^{x_{h}}\right)\left(1-e^{-x_{h}}\right)\right]}^{-1}dx_{h}$$

where $\alpha_{h}=\beta_{h}/{\beta^{\prime }}_{h}$ and $x_{h}=\hbar {\beta^{\prime }}_{h}\omega$

For the isothermal cooling process ($3^{\prime }\text{-}4^{\prime }$ branch), according to Equation (10), the amount of exhausting heat is

(16) $$Q_{c}=Q_{3^{\prime }4^{\prime }}=-\frac{F\left(n_{1},n_{2}\right)}{{\beta^{\prime }}_{c}}$$

According to Equation (12), the corresponding consumed time is similar to Equation (15) and is given by

(17) $$\tau_{3^{\prime }4^{\prime }}=\int_{\ln\left(\left(n_{2}+1\right)/n_{2}\right)}^{\ln\left(\left(n_{1}+1\right)/n_{1}\right)}{\left[e^{q\alpha_{c}x_{c}}\left(e^{\alpha_{c}x_{c}}-e^{x_{c}}\right)\left(1-e^{-x_{c}}\right)\right]}^{-1}dx_{c}$$

where $\alpha_{c}=\beta_{c}/{\beta^{\prime }}_{c}$, $x_{c}=\hbar {\beta^{\prime }}_{c}\omega$.

#### 2.4.1. The Output Power and Thermal Efficiency of QHE

An endoreversible Carnot cycle in QHE undergoes two isothermal branches and two adiabatic branches. In the entire cycle, it is assumed that the time consumed in adiabatic branches is negligible [37,38], so the total cycle period is $\tau =\tau_{1^{\prime }2^{\prime }}+\tau_{3^{\prime }4^{\prime }}$. According to Equations (14) and (16), the output work in the whole cycle is

(18) $$W=Q_{h}-Q_{c}=\left(\frac{1}{{\beta^{\prime }}_{h}}-\frac{1}{{\beta^{\prime }}_{c}}\right)F\left(n_{1},n_{2}\right)$$

The power and efficiency of QHE are given by, respectively,

(19) $$P=\frac{W}{\tau }=\frac{1}{\tau }\left(\frac{1}{{\beta^{\prime }}_{h}}-\frac{1}{{\beta^{\prime }}_{c}}\right)F\left(n_{1},n_{2}\right)$$

(20) $$\eta =\frac{W}{Q_{h}}=1-\frac{{\beta^{\prime }}_{h}}{{\beta^{\prime }}_{c}}$$

#### 2.4.2. The Operating Condition at the Classical Limit

Since the cycle period in Equations (15) and (17) cannot be evaluated, an explicit formulation has not been obtained in the general case [38]. However, it can be simplified under some specific conditions. When the system temperatures, including hot reservoir (HR), cold reservoir (CR) and WM, are high enough, it approximates to the classical limit—that is, ℏωβ≪1. Therefore, the cycle period in Equations (15) and (17) is expanded to second order approximation and is given by

(21) $$\tau =\frac{n_{2}-n_{1}}{2a}\left(\frac{1}{\alpha_{c}-1}-\frac{1}{\alpha_{h}-1}\right)$$

where $\alpha_{h}=\beta_{h}/{\beta^{\prime }}_{h}$ and $\alpha_{c}=\beta_{c}/{\beta^{\prime }}_{c}$.

The power is simplified and is rewritten as

(22) $$P=\frac{2aF_{cl}\left(n_{1},n_{2}\right)}{n_{2}-n_{1}}\cdot \frac{\alpha_{h}/\beta_{h}-\alpha_{c}/\beta_{c}}{1/\left(\alpha_{c}-1\right)-1/\left(\alpha_{h}-1\right)}$$

where $F_{cl}\left(n_{1},n_{2}\right)=\ln\left(n_{2}/n_{1}\right)$.

At the classical limit, it can be proved that the MPO is obtained when the ratios of temperatures meet $\alpha_{h}=\left(1+\gamma \right)/2$ and $\alpha_{c}=\left(1+\gamma \right)/\left(2\gamma \right)$, where $\gamma =\sqrt{\beta_{h}/\beta_{c}}$. Therefore, the operating temperatures of the hot WM and the cold WM in the isothermal branches are, respectively,

(23) $${\beta^{\prime }}_{h}=\frac{2\beta_{h}}{1+\gamma }$$

(24) $${\beta^{\prime }}_{c}=\frac{2\gamma \beta_{c}}{1+\gamma }$$

Substituting Equations (23) and (24) into Equations (22) and (20) yields, respectively,

(25) $$P_{\max}=\frac{aF_{cl}\left(n_{1},n_{2}\right)}{2\left(n_{2}-n_{1}\right)}\cdot \frac{{\left(\sqrt{\beta_{c}}-\sqrt{\beta_{h}}\right)}^{2}}{\beta_{c}\beta_{h}}=\frac{aF_{cl}\left(n_{1},n_{2}\right)}{2\left(n_{2}-n_{1}\right)}\cdot \frac{{\left(1-\gamma \right)}^{2}}{\beta_{h}}$$

(26) $$\eta_{\max P}=1-\gamma$$

Equations (25) and (26) are the MPO and the corresponding efficiency of one-stage quantum harmonic HE at classical limit. The corresponding operating time and amounts of exchange heat with two reservoirs are given by, respectively,

(27) $$\tau_{\max P}=\frac{\left(n_{2}-n_{1}\right)\left(1+\gamma \right)}{a\left(1-\gamma \right)}$$

(28) $$Q_{h,\max P}=\frac{F_{cl}}{2}\cdot \frac{1+\gamma }{\beta_{h}}$$

(29) $$Q_{c,\max P}=\frac{F_{cl}}{2}\cdot \frac{1+\gamma }{\beta_{h}}\gamma$$

Under the condition of the MPO, the work done by WM in a whole-cycle period is

(30) $$W_{\max P}=\frac{F_{cl}}{2}\cdot \frac{1-\gamma^{2}}{\beta_{h}}$$

The above results were first obtained by Geva and Kosloff [38].

### 2.5. The Simplified Combined Cycle for Multi-Stage Endoreversible Carnot QHE

**Figure 2:**
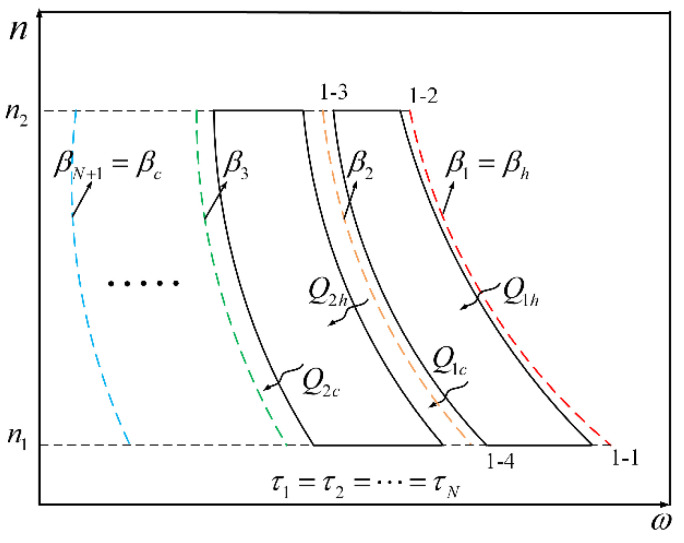
The diagram of multi-stage Carnot cycle in a quantum harmonic oscillators system.

To obtain the performance characteristics of the combined cycle Carnot QHE, this paper starts with a simplified combined model of a multi-stage cycle. It is assumed that each stage sub-cycle of the combined cycle runs at MPO. As shown in Figure 2, there are N stages in total between HR and CR. At the kth-stage cycle, the temperature of HR is $\beta_{k}$, and the temperature of CR is $\beta_{k+1}$. It is assumed that the interstage temperature, serving as the CR at the previous stage and HR at the next stage, is constant. The absorbing heat is $Q_{hk}$, the exhausting heat is $Q_{ck}$, and the work is $W_{k}$.

For the combined cycle, QHE, each stage HE is affected by an adjacent stage HE. The period of each sub-cycle impacts the synchronization of the combined cycle, and the exhausting heat rate of the sub-cycle at previous stage affects absorbing heat rate of the sub-cycle at the next stage. Due to the heat transfer characteristic in a quantum regime, two types of the above conditions, the cycle period and conservation of interstage heat current, cannot simultaneously meet in a combined cycle. Therefore, the alternative condition can ensure sustainable operation for a combined QHE.

Generally speaking, the combined forms of QHE are determined by two types of constraints, including the cycle period and interstage heat current. The first combined form, called the constraint of cycle period, demands that the period of each sub-cycle is guaranteed to be the same, i.e., the periods of each sub-cycle are equal to each other. The second combined form, called constraint of interstage heat current, demands that the exhausting heat rate of the previous stage is equal to the absorbing heat rate of the next stage. Therefore, the output performance of the combined QHE will be respectively analyzed in this paper under the two types of constraint conditions.

#### 2.5.1. The Constraint of Cycle Period

According to above analysis, the synchronization of multi-stage combined cycle can be ensured if the constraint condition is the cycle period, i.e., the cycle period of each stage must be equal to each other. Therefore $\tau_{k}=\tau_{k+1},k=1,2,\cdots ,N-1$, using Equation (27), yields

(31) $$\gamma_{1}=\gamma_{2}=\cdots =\gamma_{N}$$

where $\gamma_{k}=\beta_{k}/\beta_{k+1},k=1,2,\cdots ,N-1$.

Combining with $\gamma_{1}\gamma_{2}\cdots \gamma_{N}=\gamma =\sqrt{\beta_{h}/\beta_{c}}$ and Equation (31), the ratio of the temperatures of the two reservoirs at each stage can be denoted as

(32) $$\gamma_{k}=\gamma^{1/N}$$

According to Equation (28), the amount of absorbing heat at the kth-stage sub-cycle is

(33) $$Q_{hk}=\frac{F_{cl}}{2}\cdot \frac{1+\gamma_{k}}{\beta_{k}}$$

Since each sub-cycle runs at MPO, its efficiency is $\eta_{k}=1-\gamma_{k}$. Therefore, the output work of sub-cycle at k-stage is given by

(34) $$W_{k}=Q_{hk}\eta_{k}=\frac{F_{cl}}{2}\cdot \frac{1-\gamma_{k}{}^{2}}{\beta_{k}}$$

The total work of the N-stage combined cycle is

(35) $$W_{total}=\sum_{k=1}^{N}W_{k}=\sum_{k=1}^{N}\frac{F_{cl}}{2}\cdot \frac{1-\gamma_{k}{}^{2}}{\beta_{k}}=\frac{F_{cl}}{2}\cdot \frac{1-\gamma_{1}{}^{2}}{\beta_{h}}\cdot f(N)$$

where $f(N)=\sum_{k=1}^{N}\beta_{h}/\beta_{k}$.

The function $f(N)=\sum_{k=1}^{N}\beta_{h}/\beta_{k}$ can be rewritten as

(36) $$f(N)=\sum_{k=1}^{N}\frac{\beta_{h}}{\beta_{k}}=\frac{\beta_{h}}{\beta_{1}}+\frac{\beta_{h}}{\beta_{2}}+\cdots +\frac{\beta_{h}}{\beta_{m}}=\frac{\beta_{h}}{\beta_{1}}+\frac{\beta_{h}}{\beta_{1}}\cdot \frac{\beta_{1}}{\beta_{2}}+\cdots +\frac{\beta_{h}}{\beta_{1}}\cdot \frac{\beta_{1}}{\beta_{2}}\cdot \frac{\beta_{2}}{\beta_{3}}\cdots \frac{\beta_{N-1}}{\beta_{N}}$$

Using $\beta_{h}/\beta_{1}=1$, $\gamma_{k}=\beta_{k}/\beta_{k+1}$ and $\gamma_{1}=\gamma_{2}=\cdots =\gamma_{N}$, Equation (36) is rewritten as

(37) $$f(N)=1+\gamma_{1}{}^{2}+\gamma_{1}{}^{2}\cdot \gamma_{2}{}^{2}+\cdots +\gamma_{1}{}^{2}\cdot \gamma_{2}{}^{2}\cdot \gamma_{3}{}^{2}\cdots \gamma_{N-1}{}^{2}=1+\gamma_{1}{}^{2}+\gamma_{1}{}^{4}+\cdots +\gamma_{1}{}^{2(N-1)}=\frac{1-\gamma_{1}{}^{2N}}{1-\gamma_{1}{}^{2}}$$

Combined with Equations (32), (35) and (37), the total work of the multi-stage combined Carnot cycle QHE is equal to

(38) $$W_{total}=\frac{F_{cl}}{2}\cdot \frac{1-\gamma_{1}{}^{2}}{\beta_{h}}\cdot \frac{1-\gamma_{1}{}^{2N}}{1-\gamma_{1}{}^{2}}=\frac{F_{cl}}{2}\cdot \frac{1-\gamma^{2}}{\beta_{h}}$$

The result is the same as that of Equation (30). It can be found that, in the same temperature range, N does not affect the total output work of a multi-stage combined Carnot cycle QHE. The total amounts of absorbing heat in the entire cycle are equal to that of the sub-cycle at the first stage, and is given by

(39) $$Q_{h}=Q_{h1}=\frac{F_{cl}}{2}\cdot \frac{1+\gamma_{1}}{\beta_{1}}=\frac{F_{cl}}{2}\cdot \frac{1+\gamma_{1}}{\beta_{h}}$$

According to constraint condition, the cycle period of each stage is equal to the cycle period of the first stage. According to Equations (27) and (31), the period of combined cycle is

(40) $$\tau_{comb}=\tau_{1}=\frac{\left(n_{2}-n_{1}\right)\left(1+\gamma_{1}\right)}{a\left(1-\gamma_{1}\right)}$$

Combining with Equations (38)–(40), the overall power and thermal efficiency of simplified combined cycle QHE with the constraints of the cycle period are, respectively,

(41) $$P_{comb}=\frac{W_{total}}{\tau_{comb}}=\frac{aF_{cl}}{2\left(n_{2}-n_{1}\right)}\cdot \frac{{\left(1-\gamma_{1}\right)}^{2}}{\beta_{h}}\cdot f(N)=\frac{aF_{cl}}{2\left(n_{2}-n_{1}\right)}\cdot \frac{1-\gamma^{2}}{\beta_{h}}\cdot \frac{1-\gamma^{1/N}}{1+\gamma^{1/N}}$$

(42) $$\eta_{comb}=\frac{W_{total}}{Q_{h}}=\left(1-\gamma_{1}\right)\cdot f(N)=\frac{1-\gamma^{2}}{1+\gamma^{1/N}}$$

It can be known from the expressions that N=1 corresponds the result of a single-stage cycle, which is consistent with Equations (25) and (26).

#### 2.5.2. The Constraint of Interstage Heat Current

According to the above analysis, there is no heat loss among the sub-cycle if the constraint condition is interstage heat current. In other words, the exhausting heat rate at the previous sub-cycle is equal to the absorbing heat rate at the next sub-cycle, i.e., $Q_{c,k}/\tau_{k}=Q_{h,k+1}/\tau_{k+1},k=1,2,\cdots ,N-1$. Therefore, using Equations (27)–(29), the constraint condition is rewritten as

(43) $$\frac{1-\gamma_{k}}{\beta_{k}}\gamma_{k}=\frac{1-\gamma_{k+1}}{\beta_{k+1}}$$

Due to $\gamma_{k}=\sqrt{\beta_{k}/\beta_{k+1}}$, Equation (43) can be rewritten as

(44) $$\frac{1-\gamma_{k}}{\gamma_{k}}=1-\gamma_{k+1}$$

Therefore, the term ($1/\left(\gamma_{k}-1\right)$) is an arithmetic sequence, i.e.,

(45) $$\frac{1}{\gamma_{k+1}-1}-\frac{1}{\gamma_{k}-1}=\frac{\gamma_{k}}{\gamma_{k}-1}-\frac{1}{\gamma_{k}-1}=1$$

According to the characteristic of arithmetic sequence, the ratio of temperature of two reservoirs at a k-stage cycle is

(46) $$\gamma_{k}=\frac{1+k\left(\gamma_{1}-1\right)}{1+\left(k-1\right)\left(\gamma_{1}-1\right)}$$

Using $\gamma_{1}\gamma_{2}\cdots \gamma_{N}=\gamma =\sqrt{\beta_{h}/\beta_{c}}$, the ratio of temperatures of two reservoirs at the first stage cycle is

(47) $$\gamma_{1}=\frac{\gamma -1}{N}+1$$

Assuming that the absorbing heat rate at the first-stage is ${\dot{Q}}_{1}$, and $\gamma_{0}=1$, then the absorbing heat rate at the kth-stage is

(48) $${\dot{Q}}_{k}={\dot{Q}}_{1}\prod_{j=1}^{k}\gamma_{j-1}$$

The corresponding power at the kth-stage is

(49) $$P_{k}={\dot{Q}}_{1}\left(1-\gamma_{k}\right)\cdot \prod_{j=1}^{k}\gamma_{j-1}$$

The efficiency of a simplified combined cycle with the constraint of interstage heat current is

(50) $$\eta_{comb}=\frac{\sum_{k=1}^{N}P_{k}}{{\dot{Q}}_{1}}=\sum_{k=1}^{N}\left(1-\gamma_{k}\right)\cdot \prod_{j=1}^{k}\gamma_{j-1}=1-\prod_{k=1}^{N}\gamma_{k}=1-\gamma$$

According to Equations (25) and (44), the total power at the kth-stage sub-cycle is

(51) $$P_{k}=\frac{aF_{cl}}{2\left(n_{2}-n_{1}\right)}\cdot \frac{{\left(1-\gamma_{k}\right)}^{2}}{\beta_{k}}=\frac{aF_{cl}}{2\left(n_{2}-n_{1}\right)}\cdot \frac{{\left(1-\gamma_{k-1}\right)}^{2}}{\beta_{k}\gamma_{k-1}{}^{2}}$$

Due to $\beta_{k}\gamma_{k-1}{}^{2}=\beta_{k-1}$, Equation (51) can be rewritten as

(52) $$P_{k}=\frac{aF_{cl}\left(n_{1},n_{2}\right)}{2\left(n_{2}-n_{1}\right)}\cdot \frac{{\left(1-\gamma_{k-1}\right)}^{2}}{\beta_{k-1}}=P_{k-1}$$

Using Equation (25), the total power of the combined cycle is

(53) $$P_{comb}=\sum_{k=1}^{N}P_{k}=N\cdot P_{1}=N\cdot \frac{aF_{cl}}{2\left(n_{2}-n_{1}\right)}\cdot \frac{{\left(1-\gamma_{1}\right)}^{2}}{\beta_{1}}=\frac{aF_{cl}}{2\left(n_{2}-n_{1}\right)}\cdot \frac{{\left(1-\gamma \right)}^{2}}{\beta_{h}N}$$

It can be known from Equations (50) and (53) that, N=1 responds the result of a single-stage cycle, which is consistent with Equations (25) and (26).

### 2.6. The General Combined Cycle for Multi-Stage Endoreversible Carnot QHE

For the sake of simplification, the above analysis assumes that each stage of the combined QHE runs at the state of the MPO. This condition is very ideal. In practice, it is not guaranteed that each sub-stage of combined QHE always runs at the state of the MPO. Therefore, it needs to establish a general combined cycle for multi-stage endoreversible Carnot QHE. In this section, a two-stage combined QHE is taken as an example to optimize the output power. The method is similar when applied to optimize other multi-stage combined QHEs.

According to Equation (22), the output power of a two-stage endoreversible combined QHE can be expressed as

(54) $$P_{\mathrm{comb}}=P_{1}+P_{2}=\frac{1}{\tau_{1}}\left(\frac{1}{{\beta^{\prime }}_{1h}}-\frac{1}{{\beta^{\prime }}_{1c}}\right)F\left(n_{1},n_{2}\right)+\frac{1}{\tau_{2}}\left(\frac{1}{{\beta^{\prime }}_{2h}}-\frac{1}{{\beta^{\prime }}_{2c}}\right)F\left(n_{1},n_{2}\right)$$

where ${\beta^{\prime }}_{1h}$ and ${\beta^{\prime }}_{1c}$ are operating temperatures of the WM at the first stage QHE when it contacts the HR ($\beta_{h}$) and CR (β), respectively. ${\beta^{\prime }}_{2h}$ and ${\beta^{\prime }}_{2c}$ are operating temperatures of the WM at the second stage HE when it contacts the HR (β) and CR ($\beta_{c}$), respectively.$\tau_{1}$ and $\tau_{2}$ are the corresponding periods of the first and second stages, respectively.

When the multi-stage combined QHE operates with the constraint of cycle period, i.e., $\tau_{1}=\tau_{2}$, the constraint condition is written as

(55) $$\frac{{\beta^{\prime }}_{1c}}{\beta -{\beta^{\prime }}_{1c}}-\frac{{\beta^{\prime }}_{1h}}{\beta_{h}-{\beta^{\prime }}_{1h}}=\frac{{\beta^{\prime }}_{2c}}{\beta_{c}-{\beta^{\prime }}_{2c}}-\frac{{\beta^{\prime }}_{2h}}{\beta -{\beta^{\prime }}_{2h}}$$

where β is the intermediate temperature.

When the multi-stage combined QHE operates with the constraint of interstage heat current, i.e., $Q_{1c}/\tau_{1}=Q_{2h}/\tau_{2}$, the constraint condition is written as

(56) $${\beta^{\prime }}_{1c}\left(\frac{{\beta^{\prime }}_{1c}}{\beta -{\beta^{\prime }}_{1c}}-\frac{{\beta^{\prime }}_{1h}}{\beta_{h}-{\beta^{\prime }}_{1h}}\right)={\beta^{\prime }}_{2h}\left(\frac{{\beta^{\prime }}_{2c}}{\beta_{c}-{\beta^{\prime }}_{2c}}-\frac{{\beta^{\prime }}_{2h}}{\beta -{\beta^{\prime }}_{2h}}\right)$$

According to the constraint condition in Equations (55) and (56), two Lagrange functions are introduced, respectively,

(57) $$L_{T}=P_{\mathrm{com}}+\lambda \left[\left(\frac{{\beta^{\prime }}_{1c}}{\beta -{\beta^{\prime }}_{1c}}-\frac{{\beta^{\prime }}_{1h}}{\beta_{h}-{\beta^{\prime }}_{1h}}\right)-\left(\frac{{\beta^{\prime }}_{2c}}{\beta_{c}-{\beta^{\prime }}_{2c}}-\frac{{\beta^{\prime }}_{2h}}{\beta -{\beta^{\prime }}_{2h}}\right)\right]$$

(58) $$\;L_{q}=P_{\mathrm{com}}+\lambda \left[{\beta^{\prime }}_{1c}\left(\frac{{\beta^{\prime }}_{1c}}{\beta -{\beta^{\prime }}_{1c}}-\frac{{\beta^{\prime }}_{1h}}{\beta_{h}-{\beta^{\prime }}_{1h}}\right)-{\beta^{\prime }}_{2h}\left(\frac{{\beta^{\prime }}_{2c}}{\beta_{c}-{\beta^{\prime }}_{2c}}-\frac{{\beta^{\prime }}_{2h}}{\beta -{\beta^{\prime }}_{2h}}\right)\right]$$

The temperature $\beta_{h}$ and $\beta_{c}$ is given. Using $\partial L/\partial {\beta^{\prime }}_{1h}=0$,$\partial L/\partial {\beta^{\prime }}_{1c}=0$,$\partial L/\partial {\beta^{\prime }}_{2h}=0$, $\partial L/\partial {\beta^{\prime }}_{2c}=0$, and constraint condition Equations (53) or (54), it can get the optimal relations of $P_{\mathrm{com}}$ and β. Due to the complexity of the equations, numerical solutions will be given in the following section.

## 3. Results and Discussions

### 3.1. The Performance of the Simplified Combined Cycle QHE

The temperatures of two reservoirs are set as $\beta_{h}=1/\left(500k_{B}\right)$ and $\beta_{c}=1/\left(100k_{B}\right)$, respectively. The operating parameters and output performance of the simplified two-stage combined cycle QHE under two types of combined constraints (the constraint of cycle period is named by constraint A and the constraint of interstage heat current is named by constraint B) are listed in Table 1. The intermediate temperature is higher under the constraint of the cycle period than that under the constraint of the interstage heat current ($\beta_{h}/\gamma >4\beta_{h}/{\left(\gamma +1\right)}^{2}$). Under the constraint of interstage heat current, the output power is only half of the MPO in single-stage Carnot HE, and the efficiency remains the same. This is consistent with the conclusion of the macroscopic HE in Reference [21], which shows some similarities between the multi-stage combined QHE and the multi-stage combined classical macroscopic HE. Furthermore, the efficiency at MPO of the simplified combined cycle QHE at a classical limit under the constraint of interstage heat current can reach the Curzon–Ahlborn efficiency [96] and is equivalent to that at the low dissipation limit [97,98]. The output power and efficiency are slightly lower under the constraint of cycle period than those under the constraint of the interstage heat current. It should be noted that the powers of the two sub-stage QHEs are different. Under the constraint of the cycle period, the power of first stage HE is higher than that of the second stage QHE. Under the constraint of the interstage heat current, the output powers of the two sub-stage QHEs are equal.

**Figure 3:**
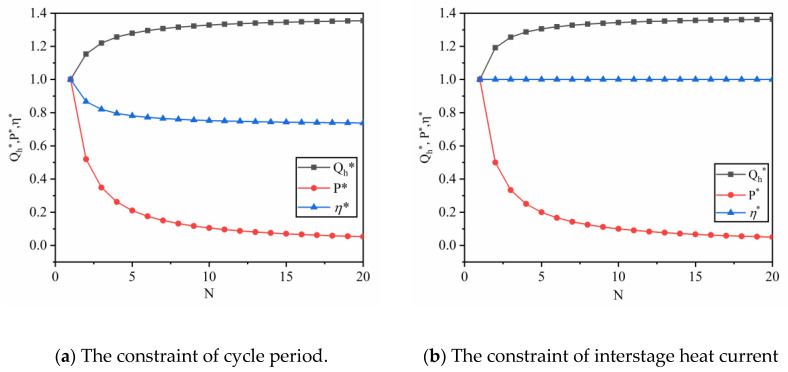
The effect of N on output performance in a multi-stage simplified combined cycle quantum heat engine (QHE).

Figure 3 shows the effect of N on the output characteristic of the combined QHE, where the ordinates are non-dimensionalized by $Q^{*}=Q_{h}(N)/Q_{h}(1)$, $P^{*}=P(N)/P(1)$ and $\eta^{*}=\eta (N)/\eta (1)$. The N has different effects on performance parameters under different combined constrains. For the constraint of cycle period, as N increases, the absorbing heat of combined QHE increases, but the total output work remains the same (the result is given in Equation (38)). The output power and thermal efficiency decrease to different levels as N increases. For the constraint of interstage heat current, the thermal efficiency of the combined QHE remains unchanged, but the output power decreases as N increases.

**Figure 4:**
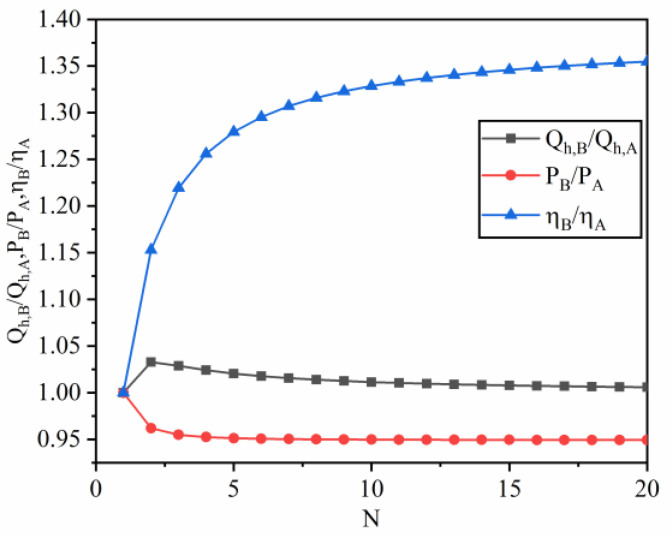
The amount of absorbing heat, output power and efficiency under constraint of interstage heat current (constraint B) versus those under the constraint of cycle period (constraint A) with different N.

The performance comparison between constraint of interstage heat current (constraint B) and constraint of cycle period (constraint A) with different N is depicted in Figure 4. In general, each combined form has its own advantages and disadvantages. The output power of the combined QHE under constraint B is slightly lower than that of the combined QHE under constraint A. And the amount of absorbing heat under constraint B is higher than that under constraint A. When increasing N, the amount of absorbing heat under constraint B is gradually equal to that under constraint A. In addition, the efficiency of the combined QHE under constraint B is higher than that of the combined QHE under constraint A. This is determined by the combined forms. The constraint of the cycle period can only guarantee the operating synchronization of each sub-cycle in QHE, but it has not been considered in terms of energy utilization. In fact, a lot of heat energy is wasted at the inter-stage of sub-cycle, resulting in a reduction in overall performance. The constraint of interstage heat current, however, does not emphasize the operating synchronization, but demands effective energy utilization. Therefore, it guarantees that the exhausting heat rate of the sub-cycle at the previous stage is exactly equal to the absorbing heat rate of the sub-cycle at the next-stage. The efficiency of the combined QHE under the constraint of interstage heat current is higher.

The role of the combined QHE is to improve efficiency and power. In fact, the essential function of the combined QHE is to increase the available temperature range between the HR and CR to enhance energy utilization. In other words, as N increases, the temperature difference between two heat reservoirs increases. To illustrate the improving extent of the output performance in combined QHE, the total performance of the combined QHE and the performance of the sub-cycle at first-stage QHE under the two types of constraints are compared. According to Equations (41), (42), (50) and (53), it is

(59) $$P_{comb}/P_{1}=\eta_{comb}/\eta_{1}=\{\begin{array}{cc} f(N) & \mathrm{Constraint}\;\mathrm{of}\;\mathrm{cycle}\;\mathrm{period}\;\\ N & \mathrm{Constraint}\;\mathrm{of}\;\mathrm{interstage}\;\mathrm{heat}\;\mathrm{current} \end{array}$$

where $P_{1}=aF_{cl}{\left(1-\gamma_{1}\right)}^{2}/\left[2\beta_{1}\left(n_{2}-n_{1}\right)\right]$ and $\eta_{1}=1-\gamma_{1}$. It is noted that the symbols ($P_{1}$,$\eta_{1}$) are different from the symbols (P(1),η(1)). The formers are performance parameters of first sub-stage of combined QHE and the latter are the performance parameters of single stage QHE.

**Figure 5:**
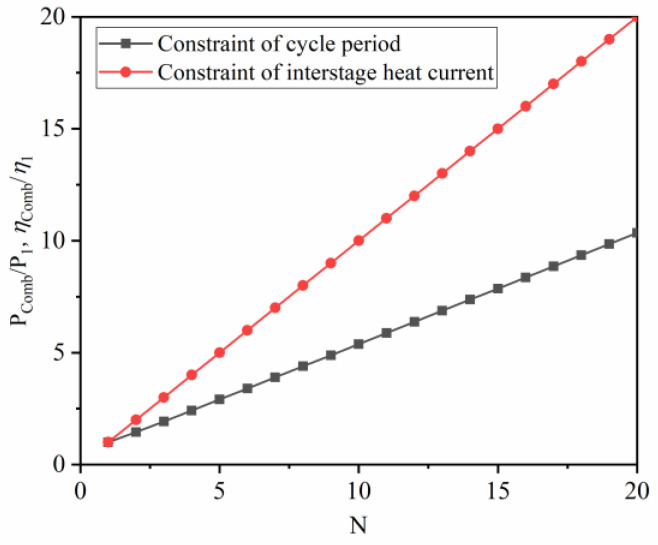
The extent of the improvement in the output power and efficiency under the two types of constraints.

It can be seen from Equation (59) that the extent of the improvement in the output power and efficiency are the same under the same constraints. As shown in Figure 5, the improvement extent of performance in the combined QHE is linearly increasing with N. Similar to the above analysis, the performance of the combined QHE under the constraint of interstage heat current is better than that of the combined QHE under the constraint of the cycle period.

### 3.2. The MPO of the General Combined Cycle QHE

The temperatures of two reservoirs are set as $\beta_{h}=1/\left(500k_{B}\right)$ and $\beta_{c}=1/\left(100k_{B}\right)$, respectively. The calculating method established in Section 2.6 is used to optimize the MPO under the two types of combined forms.

**Figure 6:**
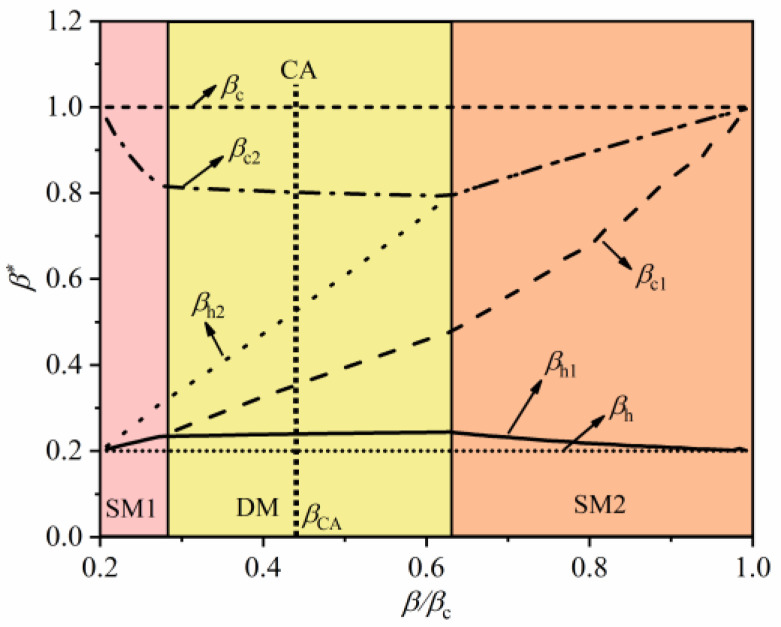
The effect of intermediate temperature on working medium (WM) temperature at maximum power output (MPO) with the constraint of the cycle period.

Figure 6 depicts the influence of the intermediate temperature on the optimal temperature of the harmonic oscillators systems under the constraint of the cycle period. In the figure, three operating modes, including the single heat engine mode at a low “temperature” (SM1), double heat engine mode (DM) and single heat engine mode at a high “temperature” (SM2), appear as intermediate temperature varies. At SM1 mode, the temperature of WM at the first stage QHE is almost constant when coupled with HR or CR. Under this condition, the output power at the first stage QHE is zero, and the total output power is mainly contributed by the second stage QHE. At the DM mode, the two sub-stage QHEs contribute to the power output. In addition, the temperature of the harmonic oscillators systems coupled with HR at first-stage QHE and the temperature of the harmonic oscillators systems coupled with CR at second-stage QHE are almost constant within the range of the DM mode. That is, the intermediate temperature mainly affects the optimal temperature of the harmonic oscillators systems coupled with CR at first-stage HE and the optimal temperature of harmonic oscillators systems coupled with HR at second-stage HE. When the intermediate temperature is greater than a certain value, it switches to the SM2 mode. In this condition, contrary to SM1 mode, the output power of combined QHE is mainly contributed to by the first stage QHE.

**Figure 7:**
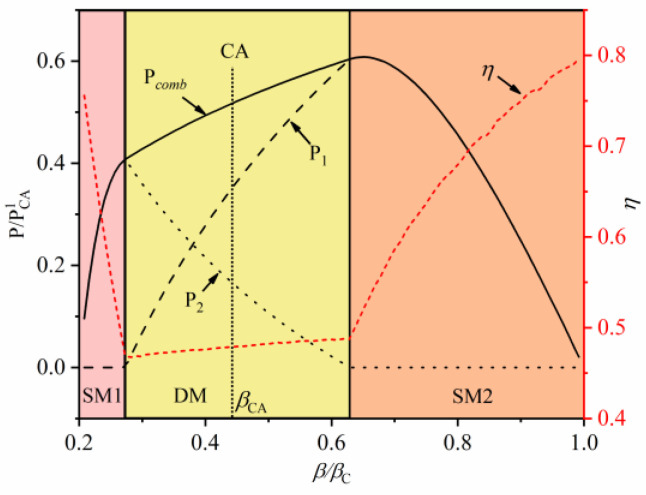
The effect of intermediate temperature on MPO and efficiency with the constraint of cycle period.

Figure 7 shows the effect of intermediate temperature on the MPO and the corresponding efficiency under the constraint of cycle period. Similar to the above analysis, three modes (SM1, DM, and SM2) of power and efficiency are presented as the intermediate temperature varies. At the SM1 mode, the output power of the second-stage QHE is equal to the total output power of combined QHE, which indicates that only the second-stage QHE works. At the DM mode, the two sub-stage QHEs can normally operate and the total power output is equal to the sum power of two sub-stage QHEs. As the intermediate temperature increases, the MPO is obtained at the junction of the DM mode and the SM2 mode. At the SM2 mode, total power starts to decrease with increases in intermediate temperature. The change trend of efficiency is opposite to that of power. The efficiency at the DM mode is smaller than those of two other modes.

It should be noted that the working conditions of the two-stage simplified combined QHE are also obtained in the optimal solution. At this condition, the intermediate temperature is $\beta_{h}=\beta_{CA}$ (the dotted line in the figures). It is worth noting that the corresponding intermediate temperature and corresponding output performance are equal to the result of N = 2 in Section 2.5.1, which illustrates the validity of the optimization method. In addition, in Figure 7, the MPOs of the combined QHE are not obtained at operation state of the simplified combined QHE. That is, the two-stage simplified combined QHE in Section 2.5.1 is only an intermediate state of the combined QHE under the DM mode. With the constraints of the cycle period, when the combined QHE deviates from the simplified combined QHE, its output power and efficiency can be higher.

**Figure 8:**
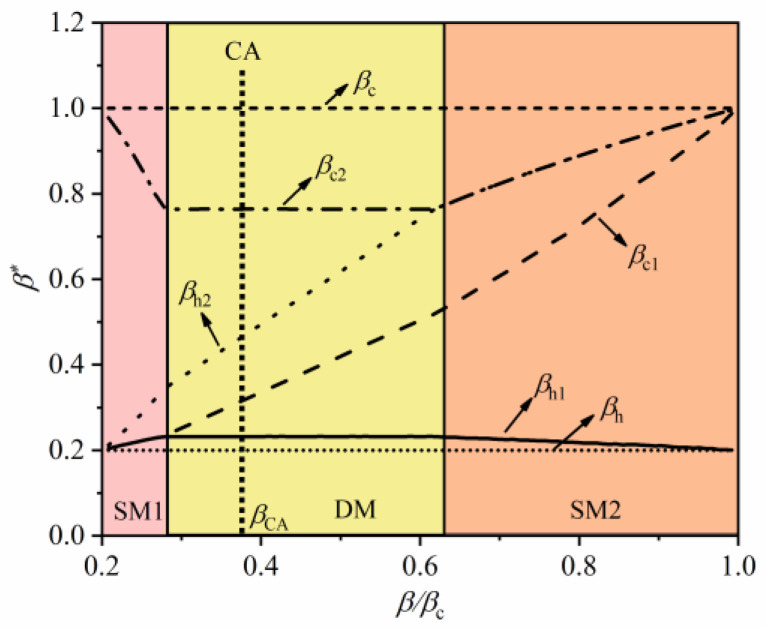
The effect of intermediate temperature on WM temperature at MPO with the constraint of interstage heat current.

**Figure 9:**
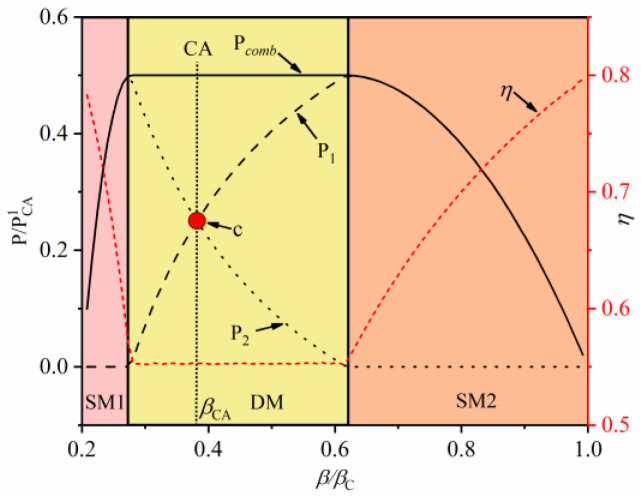
The effect of intermediate temperature on MPO and efficiency with the constraint of interstage heat current.

Figure 8 and Figure 9 depict the optimal operating parameters of the two-stage combined QHE with constraint of interstage heat current. The trend of WM temperature in Figure 8 is similar to the corresponding WM temperature in Figure 6, which indicates that the combined QHEs under the two types of constraints are similar, but the specific performances are different. Figure 9 shows the effects of intermediate temperature on MPO and efficiency under the condition of interstage heat current. Different from the constraint of the cycle period, the MPO and the corresponding efficiency of the combined QHE remain unchanged at the DM mode. When the power of a sub-stage of combined QHE decreases, and the power of the other sub-stage of combined QHE increases to compensate, keeping the overall power output unchanged.

Moreover, it can be seen that when the combined QHE runs at point c in the Figure 9, the powers of the two sub-stages of combined QHE are the same, which is consistent with the analysis in Section 3.1.

## 4. Conclusions

In this paper, the combined harmonic QHE at the classical limit is used as the study object. The powers and efficiencies of the multi-stage quantum Carnot cycle under the two types of the combined forms are studied. The main conclusions are as follows:

(1) There are two types of forms (constrains) for combining operation in QHE, that is, a constraint of the period cycle period and constraint of interstage heat current;

(2) The improvement extents of power and efficiency in the combined QHE are linearly increasing with N;

(3) Three operating modes, including single heat engine mode at low “temperature” (SM1), double heat engine mode (DM) and single heat engine mode at high “temperature” (SM2), appear in two-stage combined QHE under two combined constrains as intermediate temperature varies;

(4) For a two-stage combined QHE with constraint of cycle period, the total power at the DM mode increases as the intermediate temperature increases. The MPO is obtained at the junction of the DM mode and the SM2 mode;

(5) For a two-stage heat engine QHE with constraint of interstage heat current, the output power and efficiency are constants at the DM mode. When the power of a sub-stage of combined QHE decreases, and the powers of the other sub-stages of combined QHE increase to compensate, keeping the overall power output unchanged.

## Figures and Tables

**Table 1:** The operating parameters and output performance of the simplified combined cycle under two types of combined constraint.

Parameters	β	$Q_{h}$	P	$P_{1}/P_{2}$	η
Constraint A	$\frac{\beta_{h}}{\gamma }$	$\frac{1+\sqrt{\gamma }}{1+\gamma }Q_{h}(1)$	$\frac{1+\gamma }{1+\gamma +2\sqrt{\gamma }}P(1)$	$\frac{1}{\gamma }$	$\left(1+\gamma \right)\cdot \left(1-\sqrt{\gamma }\right)$
Constraint B	$\frac{4\beta_{h}}{{\left(\gamma +1\right)}^{2}}$	$\frac{3+\gamma }{2+2\gamma }Q_{h}(1)$	$\frac{P(1)}{2}$	1	1−γ

## Abbreviations

CR	Cold reservoir
DM	Double heat engine mode
HE	Heat engine
HR	Hot reservoir
MPO	Maximum power output
N	The number of stage
QHE	Quantum heat engine
SM1	Single heat engine mode at low “temperature”
SM2	Single heat engine mode at high “temperature”
WM	Working medium

## Nomenclature

a	Parameter of heat reservoir: $s^{-1}$
$\hat{a}$, ${\hat{a}}^{+}$	The Bosonic creation operator and annihilation operator
E	Internal energy of the harmonic oscillator system, J
$\hat{H}$	Hamiltonian
ℏ	Dirac constant (reduced Planck’s constant), J⋅s
$k_{B}$	Boltzmann constant, J/K
L	Lagrangian function
$\hat{N}$	Number operator
n	Population of the harmonic oscillators
P	Power, W
Q	Amount of heat exchange, J
$\dot{Q}$	Rate of heat flow, W
q	Parameter of heat reservoir
T	Absolute temperature, K
${\hat{V}}_{\alpha }$,${\hat{V}}_{\alpha }^{+}$	Operator and its Hermitian conjugate
W	Work, J
x	Simplified parameter ($\hbar \beta^{\prime }\omega$)
Greek symbol	
α	Ratio of temperature
β	“Temperature” ($\beta =1/\left(k_{B}T\right)$), $J^{-1}$
γ	Ratio of temperature
$\gamma_{a}$	Phenomenological positive coefficients
η	Efficiency
τ	Time/cycle period,s
ω	Thermal phonon frequency/harmonic oscillator frequency, $s^{-1}$
Subscripts	
c	Cold side
f	Terminal/final state
h	Hot side
i	Initial state
S	Working medium system
1,2,3,4	Cycle states
Superscripts	
′	Working medium
